# Removal of Algae and Algal Toxins from a Drinking Water Source Using a Two-Stage Polymeric Ultrafiltration Membrane Process

**DOI:** 10.3390/polym15234495

**Published:** 2023-11-23

**Authors:** Fan Zhang, Jianglei Xiong, Cong Zhang, Xue Wu, Yuming Tian

**Affiliations:** 1 Ecology and Environment Bureau of Huzhou, Changxing Branch, Huzhou 313100, China; 2 China Electronics System Engineering No.2 Construction Co., Ltd., Wuxi 214115, China; 3 School of Civil Engineering, Southeast University, Nanjing 210096, China; 4 Jiangsu China Electronics Innovation Environmental Technology Co., Ltd., Wuxi 214142, China

**Keywords:** algae, microcystin, ultrafiltration, polymeric UF membrane, response surface methodology

## Abstract

The release of algal toxins in algae-containing water sources poses a serious threat to drinking water safety and human health. The conventional water treatment processes of water plants have a limited ability to remove algae and algal toxins, especially algal toxins with a molecular weight (MW) of less than 1000 Da. To eliminate algal pollution from a water source, a two-stage ultrafiltration (UF) process with a large polysulfone hollow fiber membrane with a MW cut-off of 200 kDa and a small aromatic polyamide roll membrane with a MW cut-off of 1 kDa were applied after a traditional sand filter in a water treatment plant. UF operation conditions, including the operating time, pressure, and membrane flux, were investigated. With an operating pressure of 0.05–0.08 MPa, the polysulfone hollow fiber membrane removed algae effectively, as the influent algal cell concentration ranged from 1–30 cells/mL but exhibited a limited removal of algal toxins. With an operating pressure of 0.3–0.4 MPa, the elimination of microcystins (MCs) reached 96.3% with the aromatic polyamide roll membrane. The operating pressure, membrane flux, and operating time were selected as the experimental factors, and the effects on the UF efficiency to remove algal toxins and biodegradable dissolved organic carbon were investigated by the response surface methodology. The model showed that the order of influence on the membrane operating efficiency was operating pressure > membrane flux > running time. The optimal UF operating conditions were an operating pressure of 0.3 MPa, a membrane flux of 17.5 L/(m^2^·h), and a running time of 80 min.

## 1. Introduction

Algal overgrowth in drinking water sources caused by eutrophication is a common issue faced by water works in China [1,2]. Algae-related contaminants are difficult to remove by the conventional treatment processes [3,4], affecting drinking water quality adversely and reducing the operation efficiency of the water treatment plants. For example, the overgrown algae in a water source and the organic matter produced by algae might prevent the coagulation process or increase the amount of coagulants utilized in the water plants [5]. The impact of algae outbreak in raw water on drinking water safety is mainly caused by algal toxins, algal odor, and algal organic matters as the precursor of disinfection by-products [6,7]. As a widespread type of algae in fresh water, cyanobacteria generally produce highly toxic algal toxins, one of which is microcystin (MC), a strong liver tumor promoter attracting extensive attention [8], with a specific structure shown in Figure S1 in the Supplementary Materials. MCs are cyclic heptapeptide toxins with strong chemical stability and heat resistance. MCs are released into water bodies as algal cells break up and might exist for a long time in the water [9]. More than 200 isomers have been reported, and Table S1 shows several typical microcystins. Among them, microcystin-LR (MC-LR) is the most toxic and widely distributed isomer, and microcystin-RR (MC-RR) is the second most common cyanotoxin in the environment [10,11]. Traditional water treatment methods cannot effectively remove MCs from water, especially sand filtration [12,13,14]. Under practical applications, ozone and chlorine might degrade most algal toxins, but disinfection by-products (DBPs)/transformation products may also form during this process [15]. Therefore, it is urgent to develop effective methods for removing microcystins from water to address the seasonal occurrence of algal bloom or sudden outbreaks of blue-green algae in water sources.

A variety of advanced strategies, including activated carbon adsorption, ozone decomposition, photolysis, membrane filtration, and other methods, have been studied to remove algal toxins from water [16,17,18,19]. Membrane separation technologies have established themselves as essential processes of water purification and wastewater treatment. The ultrafiltration process uses an additional driving force, e.g., pressure and concentration gradients, to realize the membrane surface filtration and separation of two-component or multi-component dispersions mainly via the size sieving mechanism [20,21]. Considering the porous structural control of separation efficiency, UF membranes have been widely utilized in separation and purification processes mainly for suspended solids, colloidal particles, natural organic matters, macromolecular species, endotoxin bacteria, and even some viruses, with advantages of less energy consumption, low footprint requirements, and continuous tolerant operations [22]. Membrane manufacturing materials can be organic or inorganic. UF inorganic membranes are generally made up of ceramics, metal non-oxides, graphite, phosphates, and clays, but their high cost and modest reproducibility limit their full applicability in practice [23]. With the development of manufacturing technology and the decrease of cost, organic ultrafiltration membranes with extraordinary permeation properties and large specific surface areas have been widely used. At present, organic ultrafiltration membrane materials are mainly polymeric materials, such as acetate fiber (CA), polyvinyl chloride (PVC), polysulfone (PS), and polyvinylidene fluoride (PVDF) [24]. This is also one of the main applications of polymeric materials in water and wastewater treatment. The ultrafiltration process can be affected by various conditions, including temperature, operating pressure, influent concentration, and operation time. The optimal operation conditions should be determined when UF is applied to remove algae-related pollutants. To achieve the optimal operation conditions of UF, the response surface optimization method (RSM) can be applied, an approach of optimizing experimental conditions, which is suitable for solving problems related to nonlinear data processing [25].

The release of algal toxins in algae-containing water sources poses a serious threat to drinking water safety and human health. Algae cells and algal toxins are different pollutants, especially in size and solubility, but they usually coexist in micro-polluted water sources. During the filtration of algae-laden water, the accumulation of algal cells and their derived organic matter on the UF membrane surface and within the membrane pores could cause serious membrane fouling [26], so rejecting them by using different membranes according to their properties may be a feasible method. To investigate the removal performance of algal contaminants, especially microcystins, a two-stage UF process was applied to treat micro-polluted water after a traditional sand filter in a water treatment plant. RSM was used in this study to find the optimal operational conditions, with three factors selected during the UF process, including the operating pressure, membrane flux, and operating time. For the removal of algal toxins, the membrane flux attenuation coefficient and organic removal were selected as evaluation indicators. The objectives of this study were to assess the potential of using two-stage UF to enhance algal and algal toxin removal as an advanced treatment process in water works to ensure the safety of drinking water.

## 2. Materials and Methods

### 2.1. Materials

The two-stage UF process was composed of two different UF modules in series. The first membrane module (UF-200 kDa) was made of polysulfone hollow fiber, with a MW cut-off of 200 kDa and a membrane area of 0.2 m^2^. The operating pressure ranged from 0.045 to 0.10 MPa, generating a transmembrane flux of 50–90 L/(m^2^·h). The following spiral-wound membrane module (UF-1 kDa) was made of aromatic polyamide with a MW cut-off of 1 kDa and a membrane area of 0.8 m^2^. The operating pressure ranged from 0.1 to 0.6 MPa, generating a transmembrane flux of 10–45 L/(m^2^·h). The aromatic polyamide membrane showed a negative surface charge without modifications or in acidic solutions because of the surface carbonyl and amino functional groups [27].

The raw water samples used in the experiments were taken from the effluent of a sand filter in a water plant located in Nanjing city, where the Yangtze River water was treated as the drinking water source. The original algal cell solution was cultured in a laboratory, using the common blue-green algae species (Microcystis aeruginosa) in Taihu Lake. The blue-green algae were cultured in a light incubator for 3 weeks until the stable growth stage. The concentrated algal solutions in the stable growth period were diluted using the raw water from the sand filter and prepared as the algal solution of specific concentrations. In view of the large amount of algal toxins required by the laboratory, water with a high concentration of algae was taken from Yuantouzhu, Wuxi city, and algal toxins were extracted. Algal toxin solutions of specific concentrations (i.e., 1, 10, 30, and 50 cells/mL) were prepared using the extracted algal toxins.

### 2.2. UF Process Setup

**Figure 1:**
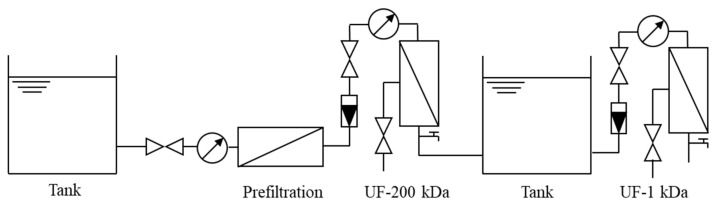
The experimental UF process.

The pilot-scale UF process is shown in Figure 1. Experimental water was pumped into a dead-end micro-filter with pore size of 0.45 µm to sieve large particles and to reduce the burden of the UF membranes. The two-stage UF processes were both performed in room temperature.

### 2.3. Analysis Methods

The counting box microscopy counting method was used to determine the algae amounts. Lugol’s solution of 10 mL was added into 1000 mL water samples for fixation in a bottle. The supernatant was removed after 48 h of standing and precipitation. Concentrated samples settled at the bottom of the bottle were stirred and 0.1 mL was taken for plate count. Average values were used after counting and calculating 3 times.

After pre-treatment, the algal toxins in the water samples were enriched using Sep-Pak C18 solid-phase extraction (SPE, Waters, Milford, MA, USA) columns and were eluted with pure methanol containing 0.1% trifluoroacetic acid (TFA). MCs were determined using Agilent 1100 high-performance liquid chromatography with a ZORBAX 80-A extended column (5 μm, 4.6 × 150 mm, Agilent, Santa Clara, CA, USA), with a mobile phase consisting of methanol and a 0.05% TFA aqueous solution and with a flow rate of 1 mL/min.

### 2.4. Response Surface Method Design

This experiment used the response surface method (RSM) to search for the optimal level of combined operating parameters for the UF-1 kDa process, i.e., how to achieve the best removal performance. Three representative influencing factors were selected for the experiment, i.e., operating pressure, membrane flux, and operating time, where the flux depended on the pressure and operating time. Considering that, in addition to the removal rate of algal toxins, the operating effect of the UF-1 kDa membrane is also related to the membrane flux and the biological stability of the filtered water (i.e., the BDOC removal rate), this study selected the membrane flux attenuation coefficient (*M*_1_), algal toxin removal (*M*_2_), and BDOC removal (*M*_3_) as evaluation indicators and determined a comprehensive evaluation index, namely, the response value *Y*_1_ (shown in Equation (1), and the calculation of *Y*_1_ is shown in the Supplementary Materials).

(1) $$Y_{1}=0.42\frac{M_{2}}{M_{2\;max}}+0.42\frac{M_{3}}{M_{3\;max}}-0.16\frac{M_{1}}{M_{1\;max}}$$

And with the help of the statistical analysis software Design Expert 7.0, the optimal process for the economic and stable operation of ultrafiltration membranes was explored. The experimental factors and levels of each group are shown in Table 1. The composition of the test water samples is shown in Table S3.

## 3. Results and Discussion

### 3.1. Overall Algal Pollutant Removal by the Two-Stage UF Process

**Figure 2:**
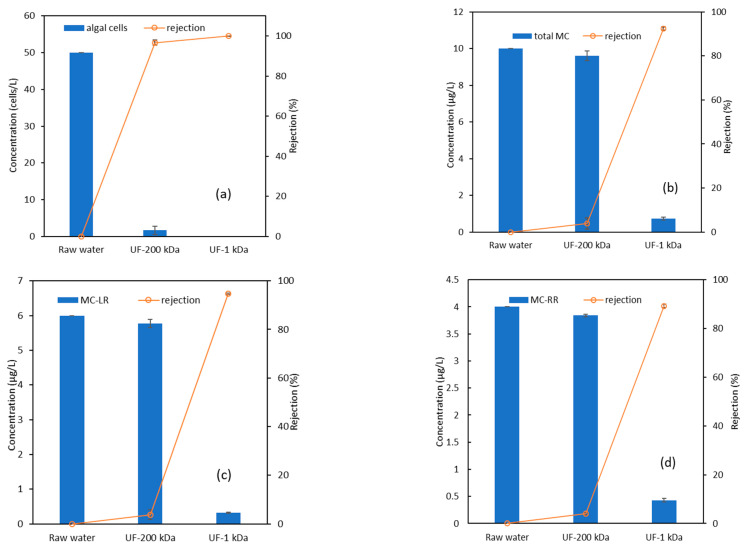
Removal of algae and algal toxins using a two-stage UF process. (**a**) Algal cells; (**b**) MCs; (**c**) MC-LR; (**d**) MC-RR.

**Figure 3:**
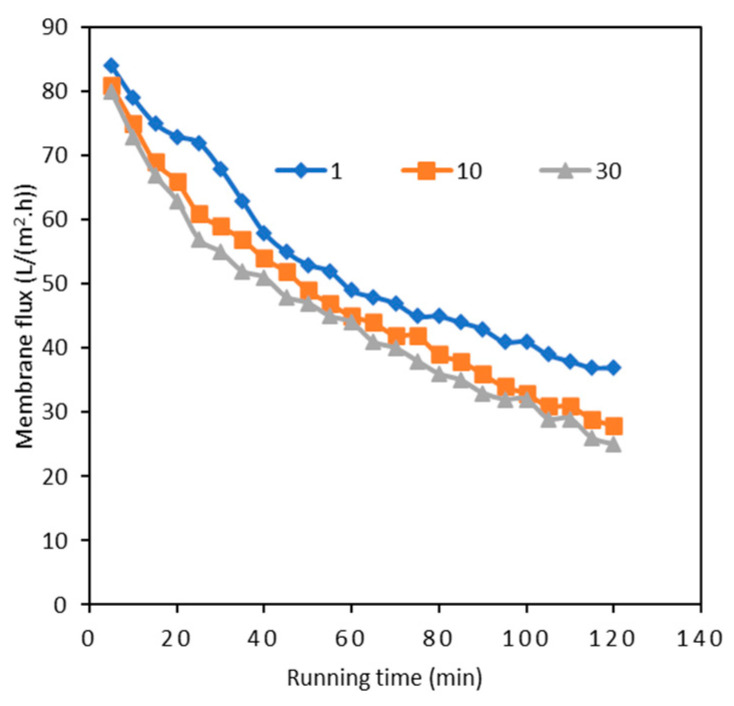
UF-200 kDa membrane flux changed with various algae concentrations.

The influent water contained 50 cells/L of algae and 10.12 μg/L of MCs (6.13 μg/L of MC-LR and 3.99 μg/L of MC-RR), and the operating pressures of the UF-200 kDa and UF-1 kDa membranes were set as constants of 0.05 and 0.3 MPa, respectively. Figure 2 shows the average removals of algal pollutants from the water during a 150 min UF operation process. The UF-200 kDa membrane rejected 96.7% of the algae cells and the rest was removed by the UF-1 kDa membrane entirely, due to the larger sizes of the algae cells (generally 1–100 µm) than those of the polymeric membrane pores. A quite limited removal of MCs was observed with the UF-200 kDa membrane (only around 3.8%). Thus, a typical UF with a large pore size could remove algae cells efficiently; yet algal toxins with molecular weights of around 1000 are difficult to remove. In addition, the membrane flux decreased rapidly because of the algae rejection. Figure 3 indicates that the UF-200 kDa membrane’s flux changes with time under an operating pressure of 0.05 MPa. For varied initial algae concentrations of 1, 10, and 30 cells/L, the flux decreased from above 80 L/(m^2^·h) to 37, 28, and 25 L/(m^2^·h), correspondingly, after running for 120 min. This rapid flux decline is caused by membrane biofouling due to the accumulation of algal cells, organic matter, and transparent exopolymer particles on the membrane surface [28]. The small amount of MC removal with the UF-200 kDa membrane was possibly due to the bio-accumulated cake layer on the membrane surface. An appropriate membrane pore size is required for an optimal operation, as it determines the dominating fouling mechanism during the retention of algal biomass. Compared with micro-filtration (MF), cake layers attached to the surface of a UF is easier to be cleaned, i.e., the MF displays a higher irreversible fouling propensity, presumably due to the internal pore plugging of submicron particles [29]. To maintain the normal function of the membrane, periodic cleaning is necessary. A UF of a higher separation effect or nanofiltration is needed to eliminate MCs from water. From Figure 2b–d, the UF-1 kDa membrane shows efficient removals of MCs with an average rejection of 93.0%. The removal of MC-LR (95.0%) was slightly higher than that of MC-RR (89.9%) with the UF-1 kDa membrane. The average concentration of residual MCs in the effluent was 0.71 μg/L, which is lower than the required concentration (1 μg/L) specified in the Standards for drinking water quality of China (GB5749-2022) [30]. Therefore, the two stages of UF processes played different roles, achieving algae and algal toxin removal, respectively.

### 3.2. Removal of Algal Toxins Using the UF-1 kDa Membrane

**Figure 4:**
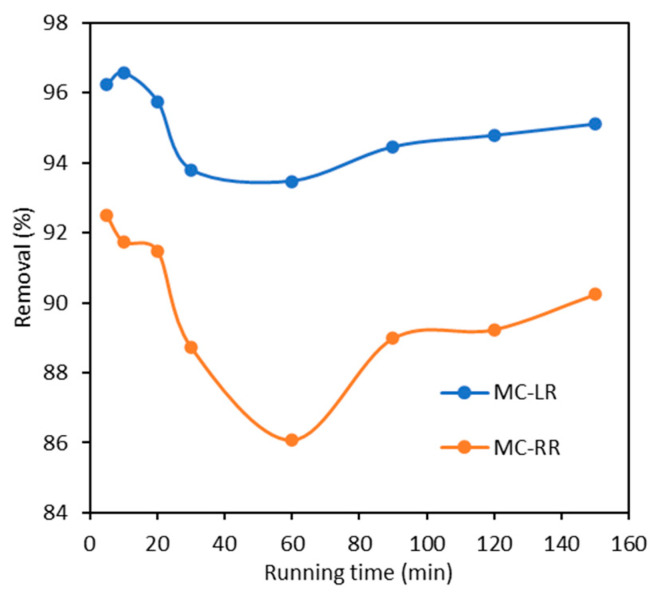
Algal toxin removal under 0.3 MPa by the UF-1 kDa membrane.

With a constant operating pressure of 0.3 MPa, the UF-1 kDa module ran for 150 min to investigate MC removal, and the removals of MC-LR and MC-RR at various times are shown in Figure 4. During the running period, the overall removals of MCs kept at above 90%. MC-LR observed higher rejections than MC-RR during the overall operation period. The negative charges on the surface of the aromatic polyamide UF-1 kDa membrane caused an electrostatic repulsion to the negatively charged MC-LR, increasing the removal compared with the neutral MC-RR [27]. The removals of MC-LR and MC-RR both show a trend of first a decrease and then an increase to a stable state. The rejection of organic pollutants during the UF process is possibly due to two reasons, including physicochemical interactions between the membrane surface and algal toxins, e.g., intermolecular forces, hydrogen bonds, and hydrophobic interactions, as well as the membrane mechanical screening [31,32]. The decrease after the initial high rejection is due to the gradual saturation of the adsorption sites on the membrane surface. And mechanical screening gradually plays a major interception role, leading to an increased rejection.

**Figure 5:**
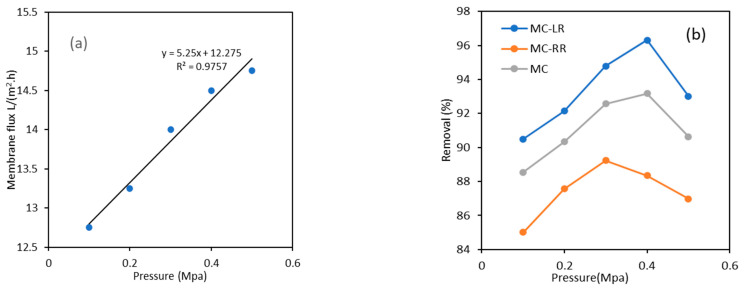
UF-1 kDa membrane’s performance under different pressures. (**a**) Membrane flux; (**b**) MC removals.

The effect of the operating pressure on the UF-1 kDa membrane’s flux after running for 100 min is shown in Figure 5. The cleaning of the UF-1 kDa membrane had a flux of higher than 30 L/(m^2^·h), and the membrane flux decreased to 14 L/(m^2^·h) with a constant pressure of 0.3 MPa after 100 min. From Figure 5a, the membrane flux shows an overall increasing trend with an increasing pressure. An approximate linear relationship was observed between the operating pressure (Δ*p*) and membrane flux (*J_w_*) as $J_{w}=5.25∆p+12.275$. As the pressure increased to 0.5 Mpa, the slope declined slightly, possibly because the concentration polarization increased the membrane resistance.

As the pressure increased, the overall removal rate of the algal toxins by UF-1 kDa membrane showed a trend of first increasing and then decreasing. However, the operating pressure for reaching the maximum removal rate was different. When the operating pressure was 0.4 MPa, the removal rate of MC-LR was the highest (96.3%) and the concentration of MC-LR in the effluent was 0.226 μg/L. When the operating pressure was 0.3 MPa, the removal rate of MC-RR was the highest (89.2%), and the concentration of MC-RR in the effluent was 0.430 µg/L. The optimal operating pressure of MC-LR was greater than that of MC-RR, determined by their charge properties. The surface charged ultrafiltration membrane had an electrostatic repulsion effect on the negatively charged MC-LR, and the occurrence of concentration polarization caused that of MC-RR to lag.

### 3.3. Analysis of MC Removal by UF Using RSM

Based on the experimental results, it was found that the operating pressure, membrane flux, and operating time have significant impacts on the membrane’s performance. Therefore, these three factors were selected as independent variables. Considering that, in addition to the removal rate of algal toxins, the operating effect of the UF-1 kDa membrane is also related to the membrane flux and the biological stability of the filtered water (BDOC removal), in this study, the removal rate of algal toxins, membrane flux, and BDOC removal rate were selected as evaluation indicators to determine the comprehensive evaluation index, which is the response value *Y*_1_. A total of 20 sets of experiments were designed to optimize the operating conditions. Based on this, a uniform design was carried out, and the data processing results are shown in Table 2. The response values *Y*_1_ ranged from 0.4096 to 0.6966. A quadratic polynomial regression model of the response value *Y*_1_ with respect to the operating pressure (*X*_1_), membrane flux (*X*_2_), and operating time (*X*_3_) was obtained as follows:

(2) $$Y_{1}=0.11205X_{1}+0.080088X_{2}+0.029123X_{3}+0.12032X_{1}X_{2}+0.004714X_{1}X_{3}-\;0.000204X_{2}X_{3}-4.11541X_{1}^{2}-0.00243X_{2}^{2}-0.000158X_{3}^{2}-1.34572$$

According to the estimated coefficients of the model, the coefficients of *X*_1_, *X*_2_, and *X*_3_ were 0.11205, 0.080088, and 0.029123, respectively. The order of effect of the influencing factors was operating pressure > membrane flux > operating time.

The results of the variance analysis on the regression model are shown in Table 3. F-regression = 29.56 > (F_0.05_ (9,5) = 4.77) and *p* < 0.001 < 0.05 indicate that the model range is highly significant. The complex correlation coefficient is 0.9638, indicating a high correlation between the measured value and the budgeted value. R^2^ = 0.9312, with a fitting degree of >90%, indicating that the model can reflect changes in response values and has small experimental errors. Therefore, this model can be used for analysis and for the prediction of *Y*_1_.

**Figure 6:**
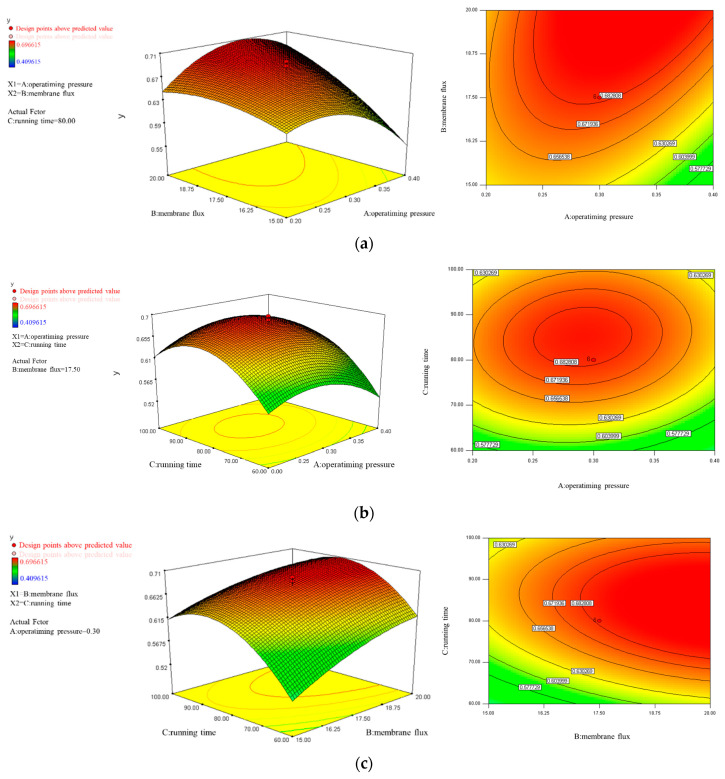
The response surface plot and contour plot analyses for the varied factors. (**a**) The effects of operating pressure and membrane flux on *Y*_1_, with an operating time of 80 min; (**b**) The effects of operating pressure and operating time on *Y*_1_ at a membrane flux of 17.5 L/(m^2^·h); (**c**) The effects of membrane flux and operating time on *Y*_1_ with a pressure of 0.3 MPa.

Figure 6 shows the response surface plot (left pictures) and contour plot analyses (right pictures). From the three-dimensional response surface plots in Figure 6a, an increase in operating pressure within a certain range at a fixed operating time of 80 min helps to increase the *Y*_1_ value, but when the *Y*_1_ value increases to a certain extent, it will show a downward trend. As the membrane flux decreases, the overall *Y*_1_ value shows a decreasing trend. From the trend chart of the contour changes, there is a maximum value of *Y*_1_, with the operating pressure ranging from 0.25 to 0.40 MPa and the membrane flux ranging from 17.5 to 20 L/(m^2^·h). From the three-dimensional response surface plots shown in Figure 6b, when the membrane flux is fixed at 17.5 L/(m^2^·h), there is a highest point on the convex surface, which is the *Y*_1_ peak. From the trend chart of the contour changes, the peak value of *Y*_1_ occurs when the operating pressure is 0.25–0.33 MPa and the operating time is 79–91 min. From the three-dimensional response surface plots shown in Figure 6c, when the fixed operating pressure is 0.30 MPa and the operating time is less than 80 min, the *Y*_1_ value increases with the increase of the operating time. However, after the operating time exceeds 95 min, the *Y*_1_ value shows an obvious downward trend. As the membrane flux decreases, the overall *Y*_1_ value also shows a gradual decreasing trend. From the trend chart of contour changes, there is a maximum value of *Y*_1_ when the membrane flux is 17–20 L/(m^2^·h) and the operating time ranges from 70 to 95 min, indicating the best operating effect.

The optimal operating conditions for the UF membrane calculated by the response surface fitting curve were considered at an operating pressure of 0.30 MPa, a membrane flux of 17.5 L/(m^2^·h), and an operating time of 80 min. These are the recommended operating parameters for ultrafiltration membrane filtration.

## 4. Conclusions

The traditional water treatment processes of water plants, e.g., coagulation, sediment, and sand filtration, have a limited ability to remove algae and algal toxins from drinking water sources. This study focused on the treatment performance of a two-stage ultrafiltration membrane combined process for the removal of algal pollutants from water. The statistical analysis software Design Expert 7.0 was used to determine the optimal conditions for UF membranes’ treatment of algae-containing water sources using the response surface analysis. The two-stage UF membrane showed a good removal effect on the algal cells and toxins under appropriate operating conditions. With an operating pressure of 0.05–0.08 MPa, the polysulfone hollow fiber membrane (MWCO of 200 kDa) removed algae effectively as the influent algal cell concentration ranged from 1–30 cells/mL but exhibited a limited removal of algal toxins. With an operating pressure of 0.3–0.4 MPa, the elimination of microcystins (MCs) reached 96.3% with the aromatic polyamide roll membrane (MWCO of 1 kDa). When using the two-stage ultrafiltration process to treat filtered water containing algae, the Design Expert software was used to simulate and analyze the comprehensive impact of the various operating parameters of the UF-1 kDa membrane on treatment efficiency and membrane flux in the following order: operating pressure > membrane flux > operating time. The optimal operating conditions for the UF-1 kDa membrane were an operating pressure of 0.30 MPa, a membrane flux of 17.5 L/(m^2^·h), and an operating time of 80 min. The two-stage UF process is a feasible, responsive strategy to combating the sudden blue-green algae outbreaks in drinking water sources. In the future, long-term operations in practical engineering should be focused on its further study, to investigate multi factors that may affect the UF’s performance, fouling of the membrane caused by bio-accumulation, and the cleaning of the membrane to enhance the UF’s performance and service life.

## Figures and Tables

**Table 1:** Response surface method design factors and levels.

Factor	Code	Level				
		−1.682	−1	0	+1	+1.682
Operating pressure (MPa)	*X*_1_	0.15	0.2	0.3	0.4	0.45
Flux (L/(m^2^·h))	*X*_2_	13.75	15	17.5	20	21.25
Operating time (min)	*X*_3_	45	60	80	100	110

**Table 2:** Response surface method design experiments and results.

Run	Code			Response Value
	*X*_1_	*X*_2_	*X*_3_	*Y*_1_
	Operating Pressure (MPa)	Flux (L/(m^2^·h))	Operating Time (min)	
1	0.2	15	100	0.579987
2	0.3	17.5	80	0.696281
3	0.4	20	100	0.64231
4	0.2	15	60	0.543629
5	0.2	20	100	0.591414
6	0.3	17.5	80	0.688964
7	0.3	21.25	80	0.695781
8	0.3	17.5	80	0.673976
9	0.3	17.5	45	0.409615
10	0.4	15	100	0.526855
11	0.3	17.5	80	0.672186
12	0.2	20	60	0.579512
13	0.3	17.5	80	0.668224
14	0.45	17.5	80	0.580424
15	0.4	15	60	0.436491
16	0.3	17.5	80	0.696615
17	0.15	17.5	80	0.609293
18	0.3	13.75	80	0.610765
19	0.3	17.5	110	0.6292
20	0.4	20	60	0.608986

**Table 3:** ANOVA for response surface quadratic model analysis of variance results.

	Quadratic Sum	Degree of Freedom	Mean Square	F-Value	*p*-Value
Model	0.12	9	0.013	29.56	<0.0001
	R = 0.9638		R^2^ = 0.9312		

## Data Availability

Data are contained within the article and Supplementary Materials.

## Supplementary Materials

The following supporting information can be downloaded at: https://www.mdpi.com/article/10.3390/polym15234495/s1, Figure S1: The molecular structure of microcystins; Text S1: The calculation of *Y*_1_; Table S1: Typical types of microcystins; Table S2: The weight values of each evaluation indicator; Table S3: Composition of tested water samples.
