# Supplementary material for: Removal of Algae and Algal Toxins from a Drinking Water Source Using a Two-Stage Polymeric Ultrafiltration Membrane Process
Source: Polymers (Basel). 2023 Nov 23;15(23):4495. doi: 10.3390/polym15234495 (PMC10708023; doi:10.3390/polym15234495)
Supplement: Supplementary file 1 [file polymers-15-04495-s001.zip › polymers-2671644-supplementary.pdf]

Supplementary materials

Removal of algae and algal toxins from drinking water source using a two-stage polymeric ultrafiltration membrane process

Fan Zhang[1], Jianglei Xiong[2*] Cong Zhang[3], Xue Wu[3], Yuming Tian[4]*

[1] *Ecology and Environment Bureau of Huzhou, Changxing Branch, Huzhou 313100, China*

[2] *Jiangsu China Electronics Innovation Environmental Technology Co.ltd, Wuxi 214142, China*

[3] *School of Civil Engineering, Southeast University, Nanjing 210096, China*

[4] *China Electronics System Engineering No.2 Construction Co.,Ltd, Wuxi 214115, China*

* Corresponding authors: F. Zhang: setsail1224@163.com; J Xiong: xiognjianglei@cese2.com

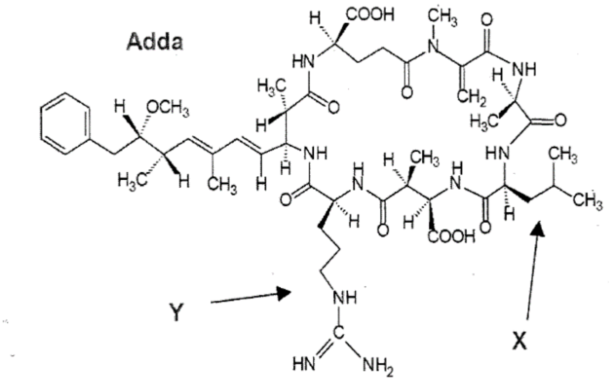

Figure S1. The molecular structure of microcystin.

Table S1 Typical types of microcystin

Type	Molecular weight	Net charge (pH=7)	Hydrophobicity
MC-LR	994	-1	Strong
MC-RR	1037	0	Weak

Text S1: The calculation of Y_1

The membrane flux attenuation coefficient M_1 is a parameter that characterizes the degree of membrane fouling. The calculation is shown as below,

$$M_1 = \frac{J_0(m^3) - J_1(m^3)}{J_0(m^3)} \times 100\% \qquad \text{(S1)}$$

Where, J_0 refers to the water flux before the experiment; J_1 is the water flux measured at the same pressure after only one cleaning after the experiment.

Considering the differences in measurement units and properties of the three evaluation indicators, this experiment adopted a comprehensive scoring method, which converted M_1, M_2, and M_3 into a comprehensive indicator Y_1. The calculation method is as follows,

$$Y_1 = n_1 \frac{M_1}{M_{1\,max}} + n_2 \frac{M_2}{M_{2\,max}} + n_3 \frac{M_3}{M_{3\,max}} \qquad \text{(S2)}$$

where, *n* represents the weight of each indicator in the comprehensive indicators.

This experiment applied the 4-scoring method to determine the weight values of three indicators. Indicators were compared with each other one by one, and were divided into four situations: (1) the very important party has 4 points, and the other party has 0 points; (2) the more important party is 3 points, and the other party is 1 point; (3) both equally important scores are 2 points; (4) self-comparison does not score. The scoring value of each indicator was divided by the sum of all scoring values as the weight. The specific scoring situation is shown in **Table S2**.

Table S2 The weight values of each evaluation indicator

Indicator	Comparison			Score	Weight
	M_1	M_2	M_3		
M_1	0	1	1	2	0.16
M_2	3	0	2	5	0.42
M_3	3	2	0	5	0.42
Sum				12	1

From **Table S2**, the calculation of Y_1 is shown in eq S3.

$$Y_1 = 0.42\frac{M_2}{M_{2\,max}} + 0.42\frac{M_3}{M_{3\,max}} - 0.16\frac{M_1}{M_{1\,max}} \qquad \text{(S3)}$$

Table S3 Composition of tested water samples

COD_{Mn} (mg/L)	UV_{254} (cm^{-1})	DOC (mg/L)	Turbidity (NTU)	BDOC (mg/L)	microcystin (μg/L)
1.89	0.079	2.9	0.13	3.33	10.125
